# Positive and negative affect as predictors of social functioning in Spanish children

**DOI:** 10.1371/journal.pone.0201698

**Published:** 2018-08-02

**Authors:** Ricardo Sanmartín, Cándido J. Inglés, María Vicent, Carolina Gonzálvez, Ángela Díaz-Herrero, José Manuel García-Fernández

**Affiliations:** 1 Department of Developmental Psychology and Didactics, University of Alicante, San Vicente del Raspeig, Alicante, Spain; 2 Department of Health Psychology, Miguel Hernandez University of Elche, Elche, Alicante, Spain; 3 Department of Evolutionary Psychology and Education, University of Murcia, Murcia, Spain; Hong Kong Polytechnic University, HONG KONG

## Abstract

The aim of this study was to analyze the relationship between affect in its two commonly used theoretical categories, positive affect (PA) and negative affect (NA), and social functioning dimensions (school performance, family relationships, peer relationships and home duties/self-care). The sample comprised 390 students of primary education aged 8–11 years (*M* = 9.39; *SD* = 1.15). The short-form of the Positive and Negative Affect Schedule for children (PANAS-C-SF) and the Child and Adolescent Social Adaptive Functioning Scale (CASAFS) were used. Student’s *t* tests indicated that those reporting high levels on all the social functioning dimensions also reported significantly higher levels of PA than peers who reported low levels; by contrast, students reporting high levels of social functioning reported significantly lower levels of NA than peers who reported low levels. Similarly, logistic regression analyses showed that an increase in PA increased probability of high levels of social functioning, and that an increase in NA decreased the probability of presenting high levels of social functioning dimensions, with the exception of school performance. These results expand the PA and NA relationship with social functioning reported in adults to Spanish children, which is potentially of interest in the fields of Education and Psychology.

## Introduction

In the literature affect has typically been divided into positive affect (PA) and negative affect (NA), and have been used extensively in the self-report mood literature, as Watson, Clark and Tellegen have noted [1]. These two affective state dimensions are mood factors that refer to feelings of enthusiasm or happiness (PA) and include aversive mood states (NA). Affect is currently of interest in the literature because its two-factor model is related to the tripartite model and helps differentiate between anxiety and depression in adults and children (NA is present in anxiety and depression, and low PA is itself a characteristic of depression) [2]. In the social context, the appearance of anxiety or depression can affect person’s emotional regulation, a key element in appropriate socialization [3]. In this sense, it is important to define social functioning as a range of skills which are used to create and develop long-term relationships that both satisfy the individual, and are essential for their psychological well-being [4]. Various studies have thus analyzed the negative implications of social function deprivation in the home, school centers, and community life [5]. Given that emotional regulation is a necessary condition for appropriate social functioning [6], addressing the relationship between affective dimensions and social functioning during childhood can help to identify the levels of affective state dimensions that are related to positive levels of such functioning. Children’s scores for social functioning are a significant element here in terms of current and future behavioral and emotional issues, not only relating to social competence but also to beliefs that have an influence on adaptive and maladaptive functioning [7], as in the case of affect.

In several self-report studies with young adults and adults (16–100 years old) from America, Australia, Portugal, Norway and Sweden, high PA has been related to better quality of life, subjective well-being and mental health [8–12], whereas high NA has been associated with low levels of quality of life and problems in daily cognitive functioning [8,13]. Also, low PA has been related to low levels of life satisfaction [14], and high PA plus low NA has been associated with less stress, uninterrupted night's rest, more energy and a more mature and persistent nature [15]. Results for children have shown similar results, with PA related to optimism and life satisfaction [16–18].

In the realm of social functioning, many studies looked at clinical young adult samples and have concluded that low social functioning is related to high levels of clinical anxiety [19–20]. In a clinical sample of children it has also been found that anxiety may be related to low levels of social functioning [21].

The relation between affect and social functioning has been investigated, in particular using clinical samples. It has been found that in young adults and adults (18–68 years old) PA has been positively related to social functioning scores [22–24] whereas NA has been related negatively [25–27]. The very few studies examining this relationship in community samples have focused on young adults and adults (18–35 years old). Results have shown that PA is positively related to social functioning scores [28] whereas NA is negatively related [29].

This brief review of the literature has shown that in general NA is negatively related to social functioning [22, 25–29] whereas PA seems to be positively related [23–24]. However, as we have noted, thus far no work has explored the relationship between affect and social functioning in children from a community sample. Testing the relation between both constructs at this stage in human development is important because analyzing social functioning scores and affect is part of the process of being able to react and prevent the emergence of behavioral and emotional problems [7, 24].

To this end, this study analyzed the relationship between affect and social functioning in primary education Spanish students aged between 8 and 11 years, with the aim of testing whether current findings in literature can be generalized to a community sample of Spanish children. The specific research objectives are: (a) to examine whether students with high marks for social functioning differ from their peers with low marks for social functioning in terms of affective scores (both PA and NA), and (b) to assess the predictive capacity of affect at high social functioning scores.

The literature reviewed above leads us to expect that:

*Hypothesis 1*: participants with high social functioning would score significantly higher in PA than their peers with low social functioning, and that PA would be a significant and positive predictor of high levels of social functioning.
*Hypothesis 2*: participants with high social functioning would score significantly lower in NA than their peers with low social functioning, and that NA would be a significant and negative predictor of high levels of social functioning.

## Materials and methods

### Sample

The sample was selected by random cluster sampling in the Spanish province of Alicante, in the region of Valencia. 10 primary schools were chosen, and from each of these 9 or 10 students were randomly selected for each of the four age groups (8, 9, 10 and 11 years old). A total of 433 students were thus recruited. However, 5% were excluded for not having the written consent of their legal tutors to participate in the research; a further 2% were excluded because they did not know the Spanish language; and 3% due to mistakes and omissions made during the tests. The final sample, then, comprised 390 students (age range = 8–11 years, *M* = 9.39; *SD* = 1.15). Of these, 47.20% were male and 52.80% female, and the age distribution was: 29.20% (8 years old), 28.50% (9 years old), 16.90% (10 years old) and 25.40% (11 years old). The χ^2^ test for uniform distribution of frequencies showed no statistical differences for the eight single-sex groups by age (χ^2^ = 4.33; *p* = .23).

Regarding the sociocultural and economic context, the data in Table 1 below are important to note.

**Table 1:** Sociocultural and economic context of participants: Academic level of the family, family structure, and number of children.

Sociocultural and economic context	Percentage of the sample
**Parental education**	
Primary studies (school graduate)	10.34%
Secondary studies (mid- and upper-level vocational training cycles or Baccalaureate)	66.77%
University studies	12.27%
Did not provide information	10.62%
**Family structure**	
Married parents	71%
Divorced or separated couples	14.6%
Other family situations	14.4%
*Parents living together but not married*	9%
*Single-parent family*	3.4%
*Did not provide information*	2%
**Number of siblings**	
Only child	13.7%
Having one sibling	55.7%
Having two siblings	18.6%
Having three or more siblings	12%

### Measures

*The 10-items Positive and Negative Affect Schedule for Children* (10-items PANAS-C [30]) is a self-report questionnaire consisting of 10 items (5 items for PA and 5 items for NA) assessed by a Likert-scale of 5 points (1 = very slightly or never; 5 = very much). PANAS-C assesses PA and NA in individuals aged between 6 and 18 years. The original scale presented appropriate values of internal consistency: .86 (PA) and .82 (NA). In the current study, the coefficients of internal consistency (Cronbach’s alpha) were .83 for PA and .81 for NA.

*Child and Adolescent Social Adaptive Functioning Scale* (CASAFS [31]) is a multidimensional self-report measure that tests social functioning in childhood and adolescence. It is formed by 24 items, these divided into 4 subscales: school performing (SP), peer relationships (PR), family relationships (FR) and home duties/self-care (HD). It uses a Likert-scale of 4 points (1 = never, 2 = sometimes, 3 = often and 4 = always). The higher the results of the subscales, the higher the person scores in social functioning. The internal consistency of the original scale ranges from .67 to.81, whereas the coefficients of internal consistency in this study were: .75 for SP, .70 for PR, .71 for FR and .73 for HD.

In order to adapt both scales to Spanish, the back-translation method was used. Initially, two specialists in English translated the two scales into Spanish independently. A native English speaker with a broad knowledge of the Spanish language then back-translated the Spanish versions of the scales into English. The new English versions of the scales were compared with the original versions and it was confirmed that the translated versions of the scales corresponded exactly to the original scales.

### Procedure

A meeting was held with the principals of each school to explain the aim of the study and to ask for their participation. A letter was then given to the students’ legal tutors including information on the tests, to be filled out and signed; this letter was included in the project information approved by the ethics committee (see below). Tests were administered during the school day (a session of approximately 30 minutes) in groups, and in the presence of a researcher, who told the students about the study before starting the session, and also emphasized the voluntary nature of the tests. A numeric code was assigned to each student to ensure anonymity. The study was approved by the Ethics Committee of the University of Alicante (File number: 20170905) and followed the standards established by the Declaration of Helsinki (1964).

### Statistical analysis

To analyze differences in the dimensions of the PANAS-C between groups with high and low social functioning, in all the dimensions (SP, PR, FR and HD), the sample was divided into groups with high social functioning (scores ≥ percentile 75) and low social functioning (scores ≤ percentile 25). Student’s *t* tests were then used to test for the existence of possible differences in the affect scores between the two groups of high and low social functioning. Moreover, the magnitude of the effect sizes of the differences found was tested with Cohen’s *d* (standardized mean difference): small (.20 - .50), moderate (.51 - .79) and large (≥ .80) [32].

The binary logistic regression method following stepwise regression, based on Wald’s statistic, was used to assess the predictive capacity of the PANAS dimensions for high social functioning scores (≥ percentile 75). Once all the statistical procedures had been performed, the odds ratio (*OR*) was used to represent the magnitude of the associations identified: scores > 1 showed a positive prediction (when affect scores increase, the probability of presenting high scores in social functioning also increases), scores < 1 indicated negative predictions (when affect scores increase, the probability of presenting high social functioning scores decreases) and scores = 1 showed no prediction [33].

For statistical analyses, the SPSS 22 program was used.

## Results

Table 2 shows the mean differences in the two categories of affect (PA and NA) for participants with high and low scores on the four social functioning dimensions (SP, PR, FR and HD). As can be seen, the Levene’s test was significant in all cases and unequal variances were assumed. Also, participants with high social functioning in all its dimensions (SP, PR, FR and HD) scored higher in PA in comparison to their peers with low scores. The magnitude of the differences in PA between the two groups showed a moderate effect size for PR (*d* = .56) and large effect sizes for SP (*d* = 1.05), FR (*d* = .89) and HD (*d* = 1.34). Regarding NA, it was found that all the individuals with high scores on all the social functioning dimensions (SP, PR, FR and HD) scored significantly lower in NA than their peers with low scores. In this sense, effect sizes for these differences were small for SP (*d* = .42), moderate for FR (*d* = .62) and large for PR (*d* = .97) and HD (*d* = .83).

**Table 2:** Means, standard deviations and effect sizes obtained from the two categories of affect for groups of high and low social functioning in all its dimensions.

		Levene’s test		Group High SF		Group Low SF		Statistical significance and magnitude of differences		
SF dimensions	Affect categories	*F*	*p*	*M*	*SD*	*M*	*SD*	*t*	*df*	*d*
SP	PA	29.15	< .001	22.13	2.78	18.25	4.43	-6.75**	102.31	1.05
	NA	27.88	< .001	7.31	3.46	9.08	4.90	2.70*	110.54	.42
PR	PA	37.71	< .001	21.09	3.17	18.95	4.35	-4.83**	221.25	.56
	NA	34.43	< .001	7.00	3.09	10.50	4.03	8.44**	230.04	.97
FR	PA	16.67	< .001	21.00	3.31	17.74	4.00	-6.89**	136.04	.89
	NA	19.38	< .001	7.63	3.33	10.00	4.25	4.76**	131.12	.62
HD	PA	142.24	< .001	21.97	2.24	17.11	4.61	-10.27**	136.95	1.34
	NA	19.28	< .001	7.03	2.66	9.50	3.25	6.69**	189.50	.83

*Note*: SF = Social Functioning; PA = Positive Affect; NA = Negative Affect; SP = School Performance; PR = Peer Relationships; FR = Family Relationships; HD = Home Duties/Self-Care

* = *p* < .05

** = *p* < .001.

Table 3 sets out the results of the binary logistic regressions. With respect to the percentage of cases correctly classified, it should be noted that this ranged from 76.5% for SP to 80% for PR. Similarly, values of the R^2^ of Nagelkerke ranged from .24 for FR to .46 for HD. On the one hand, results indicated that the PA dimension significantly and positively predicted high scores on all the social functioning dimensions (SP, PR, FR and HD); the *OR* established that for each point that the PA scores increase, the probability of presenting high levels in the social functioning dimensions increases, 34% for SP, 11% for PR, 22% for FR and 42% for HD. On the other hand, the results show that the NA dimension significantly and negatively predicted high scores in all the social functioning dimensions (PR, FR and HD), with the exception of SP. In this case, the *OR* showed that for each point that the NA scores increase, the probability of presenting high levels in the social functioning dimensions decreases, 21% for PR, 12% for FR and 18% for HD.

**Table 3:** Binary logistic regression for the probability of presenting high social functioning in terms of the two subscales of the PANAS.

SF	Affect	χ^2^	B	SE	Wald	*p*	*OR*	95% CI
SP	PA	47.95	.29	.05	35.11	< .001	1.34	1.22–1.47
	NA		-	-	-	*n*.*s*.	-	-
	Constant		-5.40	1.03	27.61	< .001	.005	
PR	PA	76.77	.10	.03	8.93	.003	1.11	1.04–1.18
	NA		-.24	.04	41.89	< .001	.79	.73-.85
	Constant		.40	.81	.25	.621	1.49	
FR	PA	60.55	.20	.04	32.72	< .001	1.22	1.14–1.31
	NA		-.12	.03	13.79	< .001	.88	.83-.94
	Constant		-1.80	.78	5.41	.020	.16	
HD	PA	121.93	.35	.05	51.21	< .001	1.42	1.29–1.56
	NA		-.21	.06	14.03	< .001	.82	.73-.91
	Constant		-4.79	1.09	19.27	< .001	.01	

*Note*: SF = Social Functioning; PA = Positive Affect; NA = Negative Affect; SP = School Performance; PR = Peer Relationships; FR = Family Relationships; HD = Home Duties/Self-Care.

## Discussion

This study seeks to analyze the relationship between the two dimensions of positive affect and negative affect (PA and NA) following the distinction by Watson et al. [1], and the social functioning dimensions according to the classification of Price et al. [31]: School Performance, Peer Relationships, Family Relationships and Home Duties and Self-Care (SP, PR, FR and HD).

The first study hypothesis was confirmed by the results obtained from a clinical sample [23–24]. High levels in social functioning scored significantly higher in PA in comparison with low levels of social functioning, and PA positively predicted high levels of all social functioning dimensions (SP, PR, FR and HD). This relationship may be explained by the association that has been found between PA and the adaptive nature of adolescents [34]. An adolescent with an adaptive personality would present good social functioning skills and thus show adaptive psychological well-being [4]. Although these results have been obtained from a community sample, and may not be directly compared with findings from other such studies, it is essential to continue investigating the relation between PA and social functioning using the PANAS. This needs to be explored in more detail, as noted by a number of researchers [24], given that it may be quite useful for clinical samples [35].

Regarding the second hypothesis, high levels of social functioning scored significantly lower in NA than low levels, and NA negatively predicted high levels of all of the social functioning dimensions (PR, FR and HD), with the exception of SP. Hence, the hypothesis has been confirmed with results obtained from the clinical [22, 25–27] and community [28–29] samples. Despite the absence of other studies considering the relation between NA and social functioning in children, these results support the use of the scores for these constructs on the Patient Reported Outcome Measurement Information System (PROMIS) in order to perform subsequent comparative studies [36]. It is important to consider both constructs as possible objects of study for future longitudinal studies, given that in clinical samples, it has been found that individuals with high levels of depression tend to be more isolated, build fewer friendships and be victimized by people around them [19–20]. All of these aspects negatively influence the development of social functioning, as observed in various studies where anxiety and depression, which are characterized by high levels of NA [2, 37], have been associated with low levels of social functioning [38]. Therefore, a useful line of future research may examine whether the reduction of NA can help to improve social functioning scores.

It is of great importance to develop treatments to improve the levels of affect that influence social functioning, thus positively impacting the development and happiness of the individual. Several studies have argued against the idea of a genetic component related to social functioning [39], but that it may be trained and improved from a psychological and educational perspective. Given that other investigations have shown how social functioning can be improved with various strategies, such as the generation of impressions about mental states of other people or the empowerment of flexibility to juxtapose multiple impressions [40–42], it would be interesting to analyze the role of PA on said improvements. Elsewhere, it has been found that low levels of PA are positively associated with depression [2, 37], which is a maladaptive construct negatively linked to social functioning [38], and that high PA can be positively related to social functioning [24]. So, considering the positive relation between PA and high levels of all the social functioning dimensions (SP, PR, FR and HD) may be a useful starting point to employ this affective dimension in future treatments to improve social functioning in the child population and to prevent the emergence of maladaptive factors such as anxiety or depression.

Despite the positive aspects of this study, it also has certain limitations. First, the analyses do not allow to establish a cause-effect relationship between the two constructs, so further studies using longitudinal data or structural equation modelling are necessary. Also, all of the data came from self-report measures and these can generate a response bias, which could be avoided in future studies by using multi-source research with diverse informants such as reports from parents, teachers and class-mates, or even by using techniques such as oral interviews with participants. On the other hand, the study has been conducted on a child community sample, so it would be interesting for future studies to examine the relation between affect and social functioning in a child clinical sample. Moreover, in order to compare these results from the Spanish sample, further studies should consider Spanish child and adolescent samples, looking at aspects such as sex and age, which are considered important in the field of affect [43–44]. Additionally, it would be useful to examine the relationship between social functioning and other variables that can affect a student’s well-being, such as school refusal or perfectionism [45–46].

Despite these limitations, this study established some initial findings on the relation between affect and social functioning in young subjects. An adaptive capacity of PA as it relates to high levels of social functioning, and a maladaptive capacity of NA in relation to low levels of social functioning, have been observed. Future works that consider these findings could lead to significant advances in the improvement of the social response of individuals, which plays an essential role in the development of psychological well-being.

## Conclusions

The results of this investigation, in accordance with the previous literature, highlight the significant and positive relation between PA and high levels of social functioning, and the significant and negative relation between NA and high levels of social functioning. On these lines, this study recommends increasing PA and decreasing NA levels in children, which can be done with the help of specific programs oriented towards improving personal development or promoting well-being, as in Fortius [47]. Additionally, during recent years it has been observed that cognitive restructuring techniques and mindfulness have also been used to increase the levels of PA [48]. These recommendations should also be considered when working with the affective dimension in the fields of Education and Psychology; PA has not only been related to anxiety, depression and to the social performance of an individual, but also has a notable impact on quality of life, subjective well-being and life satisfaction.

## Supporting information

S1 Table Data set of affect and social functioning. (XLSX)
